# Supplementary material for: The Cystic Fibrosis Transmembrane Conductance Regulator Gene (CFTR) Is under Post-Transcriptional Control of microRNAs: Analysis of the Effects of agomiRNAs Mimicking miR-145-5p, miR-101-3p, and miR-335-5p
Source: Noncoding RNA. 2023 Apr 18;9(2):29. doi: 10.3390/ncrna9020029 (PMC10146138; doi:10.3390/ncrna9020029)
Supplement: Supplementary file 1 [file ncrna-09-00029-s001.zip › ncrna-2296740-supplementary.pdf]

The Cystic Fibrosis Transmembrane Conductance Regulator Gene (CFTR) is under post-transcriptional control of microRNAs: analysis of the effects of agomiRNAs mimicking miR-145-5p, miR-101-3p, and miR-335-5p

Chiara Papi [1], Jessica Gasparello [1], Matteo Zurlo [1], Lucia Carmela Cosenza [1], Roberto Gambari [1,2] * and Alessia Finotti [1,2] *

[1] Department of Life Sciences and Biotechnology, University of Ferrara, Italy
[2] Research Center on Innovative Therapies for Cystic Fibrosis, University of Ferrara, Italy

* Correspondence: alessia.finotti@unife.it (A.F.); gam@unife.it (R.G.)
Tel.: +39-0532-974510 (A.F.); +39-0532-974443 (R.G.)

SUPPLEMENTARY MATERIALS

FIGURE S1

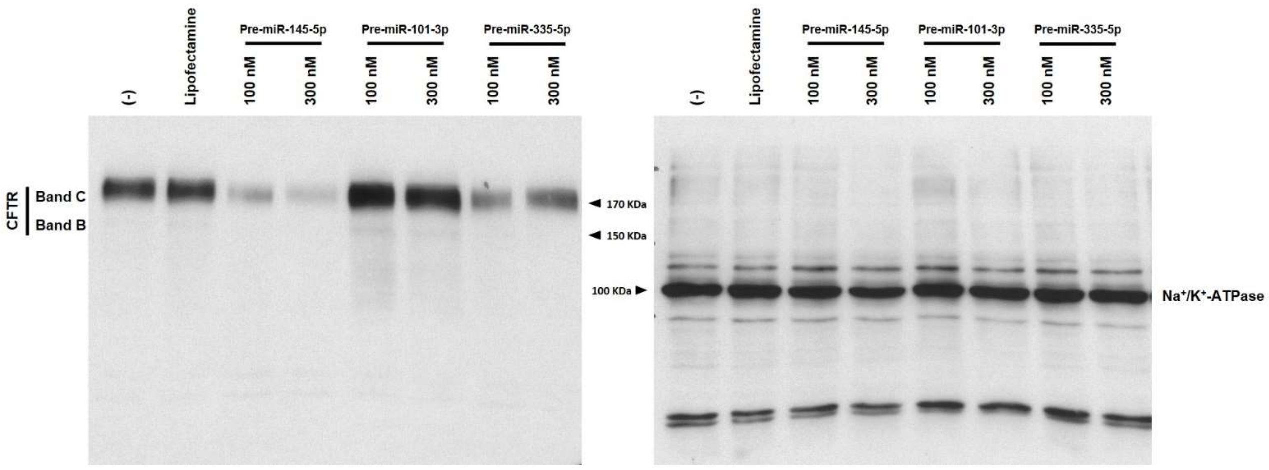

Supplementary Figure S1. Uncut version of the Western blotting gels used for data reported in Figure 2, upper part of the panel (CFTR) and Figure 2, lower part of the panel (Na^+/K^+-ATPase).

FIGURE S2

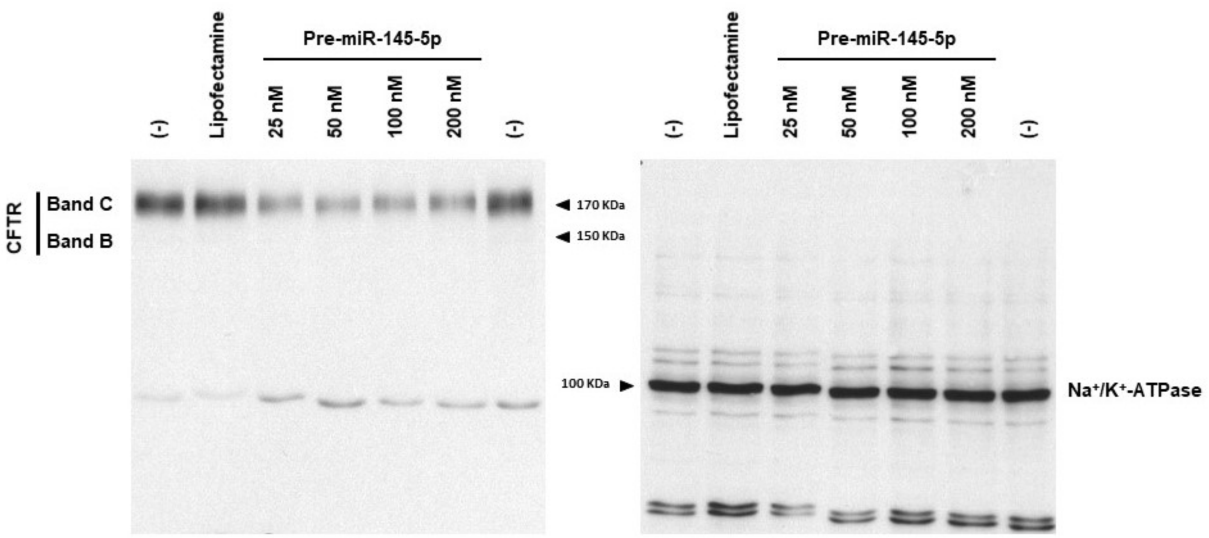

Supplementary Figure S2. Uncut version of the Western blotting gels used for data reported in Figure 3B and Figure 3C.

FIGURE S3

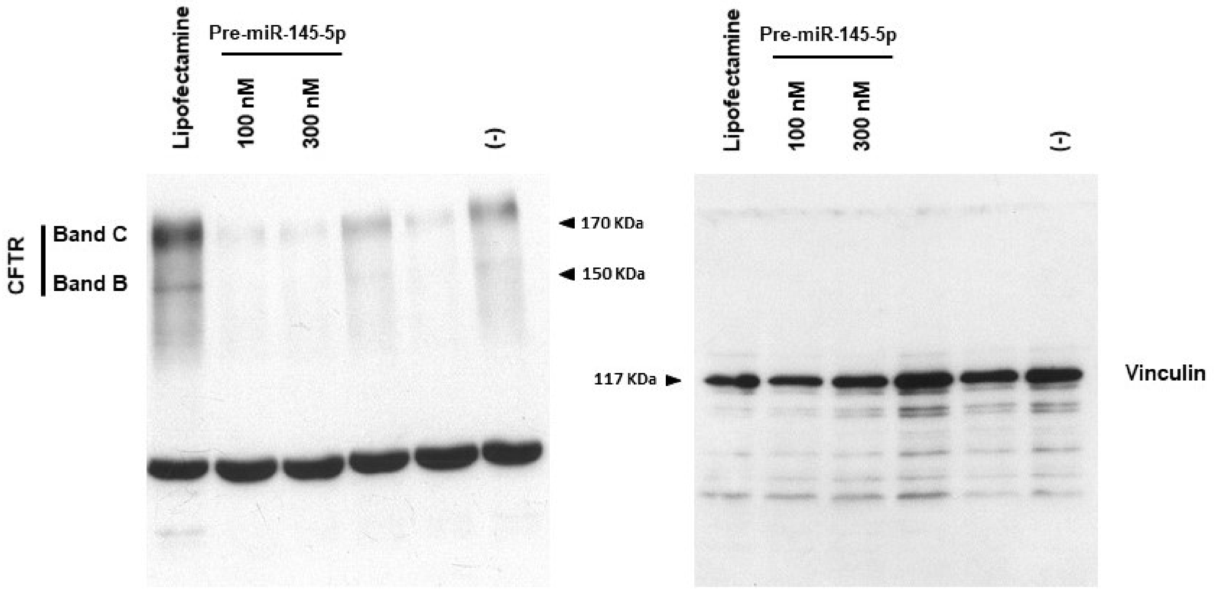

Supplementary Figure S3. Uncut version of the Western blotting gels used for data reported in panels B and C of Figure 5.
